# Vaccine Effectiveness against GP-Attended Symptomatic COVID-19 and Hybrid Immunity among Adults in Hungary during the 2022–2023 Respiratory Season Dominated by Different SARS-CoV-2 Omicron Subvariants

**DOI:** 10.3390/vaccines12050496

**Published:** 2024-05-04

**Authors:** Judit Krisztina Horváth, Gergő Túri, Katalin Krisztalovics, Katalin Kristóf, Beatrix Oroszi

**Affiliations:** 1 National Laboratory for Health Security, Epidemiology and Surveillance Centre, Semmelweis University, 1085 Budapest, Hungary; horvath.judit.krisztina@semmelweis.hu (J.K.H.); turi.gergo@semmelweis.hu (G.T.); krisztalovics.katalin@semmelweis.hu (K.K.); 2 Institute of Laboratory Medicine, Clinical Microbiology Laboratory, Semmelweis University, 1085 Budapest, Hungary; kristof.katalin@semmelweis.hu

**Keywords:** vaccine effectiveness, COVID-19, SARS-CoV-2, hybrid immunity, test-negative design, booster vaccination, reinfection, Omicron subvariants, primary care

## Abstract

Hungary provides the opportunity to evaluate the effectiveness of COVID-19 vaccination in a setting where naturally acquired immunity and hybrid immunity are likely to play a greater role due to suboptimal vaccination coverage. Methods: A test-negative study was conducted during the 2022–2023 respiratory season at the primary care level to determine the effectiveness of at least one COVID-19 booster dose in preventing medically attended symptomatic RT-PCR-confirmed SARS-CoV-2 infection in adults. Unvaccinated patients were used as a reference group. Results: A total of 247 cases and 1073 controls were included in the analysis. CVE was 56.8% (95% CI: 11.9–78.8%) in the population aged 60 years and older and 2.3% (95% CI: −50.0–36.3%) in the younger adults against COVID-19 caused by Omicron subvariants, mainly BA.5, BQ.1, and XBB.1. Self-reported COVID-19 in the 60–365 days prior to the current illness did not confer protection against reinfection without vaccination, but together with booster vaccination, it reduced the risk of COVID-19 by 63.0% (95% CI: −28.0–89.3%) and 87.6% (95% CI: 26.4–97.9%) among the 18–59 and 60+ age groups, respectively. Conclusions: CVE against COVID-19 was moderately high in the 60+ age groups. Because of the benefit of hybrid immunity, persons with previous SARS-CoV-2 infection should still be considered for vaccination campaigns.

## 1. Introduction

The genetic structure of SARS-CoV-2 is subject to constant change, which makes it capable of causing epidemics at any time of the year, and even, occasionally, pandemics. The emerging new variants of SARS-CoV-2 often have a significant selection advantage because they can evade the immune system more effectively, and the neutralizing activity of antibodies induced by previous infection or vaccination declines over time [1,2]. Protective immunity against COVID-19 is complex and involves cellular immunity as well as neutralizing antibodies. The variable effectiveness of COVID-19 vaccines against Omicron subvariants poses a challenge to understanding protection and the success of vaccination programmes [3,4].

COVID-19 vaccine effectiveness (CVE) is defined as the ability of a vaccine to reduce the risk of COVID-19 disease under routine, daily use. CVE may vary over time and across geographical areas. Therefore, it is recommended to monitor and evaluate CVE through observational epidemiological studies [5,6]. Up-to-date data on CVEs can inform the rapid development and distribution of new vaccines with modified formulations. It can also trigger timely recommendations for necessary non-pharmacological measures to prevent COVID-19 in the population if vaccine effectiveness declines.

During the 2022–2023 respiratory season, several Omicron subvariants of the SARS-CoV-2 virus were prevalent. These subvariants have been shown to have increased immune evasion and reduced vaccine effectiveness, particularly if the time since last vaccination or previous infection has increased [7]. Several studies have shown that a primary vaccination series with one or more booster doses against COVID-19, including severe cases, caused by Omicron subvariants BA.1, BA.2, BA.5, BQ.1 and XBB.1, is short-lived and provides only low to moderate effectiveness [4,8,9,10,11].

There is considerable variation in COVID-19 vaccine uptake between countries, which in turn affects the level of SARS-CoV-2 transmission. In Hungary, the COVID-19 vaccination programme started at the end of 2020, using a unique vaccine portfolio of six types of monovalent COVID-19 vaccines [12]. By week 20 of 2023, the cumulative uptake of the primary course of COVID-19 vaccination among the Hungarian population aged 18 years old and older was 71.5%, 11% lower than in the EU/EEA countries. Approximately 90% of the Hungarian population aged 18 years and over, who followed the vaccination recommendation, received their first COVID-19 vaccine booster before the third week of 2022. The coverage of the first booster in Hungary was 47.5% in week 20 of 2022, which is 18% lower than the EU/EEA average. The second booster was administered from week 1 of 2022, resulting in a coverage of 5.2%, which is 17.9% below the EU/EEA average. In Hungary, the third COVID-19 booster was administered starting from week 36 of 2022 and reached a cumulative uptake of 0.2%, which is 2.7% lower than the EU/EEA average [13]. The most commonly used booster vaccines in Hungary were mRNA vaccines [12].

Recent data on vaccine effectiveness mainly come from high-income countries where a significant proportion of the population has completed their primary vaccination, and many have also received booster doses [14,15]. Limited data are available on vaccine effectiveness in countries where natural immunity or hybrid immunity, resulting from both vaccination and natural infection, may play a greater role. Furthermore, the absolute CVE approach, which uses unvaccinated individuals as the reference group, is becoming increasingly uncommon. However, it remains of public health importance in countries where there are still substantial numbers of unvaccinated individuals. The lower vaccination coverage in Hungary, compared to the EU/EEA average, provided an opportunity to evaluate CVE, with a particular focus on prior infection and hybrid immunity.

This study aims (1) to estimate the effectiveness of the COVID-19 vaccination in preventing medically attended COVID-19 cases among individuals aged 18–59 and 60 years and older in Hungary during the 2022–2023 respiratory season, taking into account the different circulating Omicron subvariants; (2) to evaluate the protective effect of hybrid immunity against the mixture of circulating Omicron subvariants, in comparison to vaccination or natural infection alone; and (3) to investigate and assess any potential effects of testing prior to the GP consultation on CVE estimation.

## 2. Materials and Methods

### 2.1. Study Design and Study Population

As part of the ECDC-VEBIS (Vaccine Effectiveness, Burden and Impact Studies) project, we conducted a test-negative primary care case–control study, involving 68 general practitioners (GPs) from Hungary. The study period spanned from ISO week 36 in 2022 to ISO week 12 in 2023, based on symptom onset. The study protocol used in Hungary was adapted from the core protocol for ECDC studies on vaccine effectiveness against symptomatic, laboratory-confirmed SARS-CoV-2 infection in primary care settings [16].

Participants aged 18 years or older were recruited if they presented to one of the GPs with symptoms meeting the EU definition of acute respiratory infection (ARI), which includes sudden onset of symptoms and at least one of the following respiratory symptoms: cough, sore throat, shortness of breath, coryza, and a clinician’s judgement that the illness was due to an infection. All patients who met the case definition were invited to participate in the study. After providing informed consent, participants were swabbed for testing.

### 2.2. Data Collection

The GPs collected information on basic demographics, symptoms, underlying chronic conditions, and COVID-19 vaccination status using REDCap (Research Electronic Data Capture) Version 14.3.5, a web-based software platform, designed for research data collection and data management, hosted at Semmelweis University [17]. In addition, data were collected using a GP questionnaire to determine if the patient had a confirmed case of COVID-19 prior to their current illness episode, the date of their positive SARS-CoV-2 test, and the type of test used. Also, it was asked if the patient had undergone a COVID-19 test related to their current symptoms before GP consultation, the type of COVID-19 test, and whether any of the previous COVID-19 tests had given a positive result.

Information on COVID-19 vaccination status was obtained primarily from the National Immunization Registry, where there was a barrier to this, from GP records or self-report.

All SARS-CoV-2-positive samples with CT scores less than 30 underwent sequencing to determine the complete viral genome and strain. The library preparation, sequencing, and bioinformatic analysis steps were performed by iBioScience Ltd. in Pécs, Hungary. The libraries for Illumina sequencing were prepared using hybridization capture methods. Briefly, total RNA was fragmented and reverse-transcribed, and then the cDNA was end-prepped, adapter-ligated, and amplified. Finally, the hybridization capture was performed using SARS-CoV-2-specific probes. The libraries were sequenced on NovaSeq 6000 platform using 2 × 150 paired-end (PE) chemistry. Strain classification was based on the analysis of raw data, quality control, screening, removal of adaptors and primers, and determination of the whole genome and variants using Pangolin software [18]. Quality control was performed using FastQC v0.11.8 with a QV > 30 and Trim Galore v0.4.4 [19,20].

### 2.3. Patient Exclusion Criteria

Patients who did not meet the case definition were under 18 years of age, lived in a residential care facility, had a contraindication to COVID-19 vaccination, lacked information on COVID-19 vaccination status, had previous COVID-19 less than 60 days before the current onset, were swabbed for PCR more than 10 days after the onset of symptoms, or had inconclusive test results in relation to the current symptoms (previous positive and current negative SARS-CoV-2 PCR test result), and thus were excluded from the study.

### 2.4. Vaccination Definition

Participants were considered fully primary-vaccinated 14 days after receiving either a single dose of the Janssen vaccine or the second of two recommended doses of a two-dose vaccine. Participants who received a first booster dose ≥120 days after the last dose of the primary series or ≥60 days after a single dose of Janssen and received their last booster dose ≥14 days before their onset of symptoms were considered booster-vaccinated. The second booster dose had to be administered at least 120 days after the first. Participants who did not receive at least one booster dose after the primary vaccination series were excluded from the analysis.

### 2.5. Statistical Analysis

Data analysis was conducted using the Stata statistical programme 17.0 BE-Basic Edition. Patients who were laboratory-confirmed SARS-CoV-2-positive by RT-PCR were considered as COVID-19 cases, and those who tested negative for SARS-CoV-2 by RT-PCR were included in the control group. The reference group included those who had not received any dose of the COVID-19 vaccine.

We described the weekly number of cases and controls recruited by date of swab, and the corresponding number of Omicron subtypes detected from the respiratory tract of the confirmed cases, and the baseline characteristics of cases and controls.

A logistic regression model was used to estimate the odds ratio (OR) of receiving at least one COVID-19 booster compared to no vaccination. The model was adjusted for a week of symptom onset, age, sex, and the presence of at least one chronic condition, such as diabetes, immunodeficiency, lung disease, and heart disease. The CVE was calculated as (1 − OR) − 100 (%), using unvaccinated patients as the reference group, by time since vaccination (14–365, 366+ days) and by age group (18–59, 60+ years). To minimize bias from rare events, the CVE was estimated using penalized logistic regression (Firth’s penalized likelihood method) for models with fewer than 20 exposed or unexposed patients or fewer than five vaccinated cases [21].

We investigated whether previous self-reported SARS-CoV-2 infection was a potential confounding factor, since it may influence both the probability of vaccination and the development of a new infection. Additionally, the study analysed the protection provided by self-reported SARS-CoV-2 infection prior to the current episode with or without COVID-19 vaccination, in comparison to those who had neither reported prior infection nor received vaccination, with estimating stratified CVEs.

An analysis was conducted to determine if pre-consultation testing (PCT) for SARS-CoV-2 could affect CVE estimates by differentially influencing GP consultation according to vaccination status. We compared the baseline characteristics between those with and without PCT, analysed association with vaccination, and estimated stratified CVEs. Additionally, we compared the overall CVE from the original model with CVE controlled for PCT to see its potential effect.

## 3. Results

### 3.1. Study Population and Descriptive Analysis

**Figure 1:**
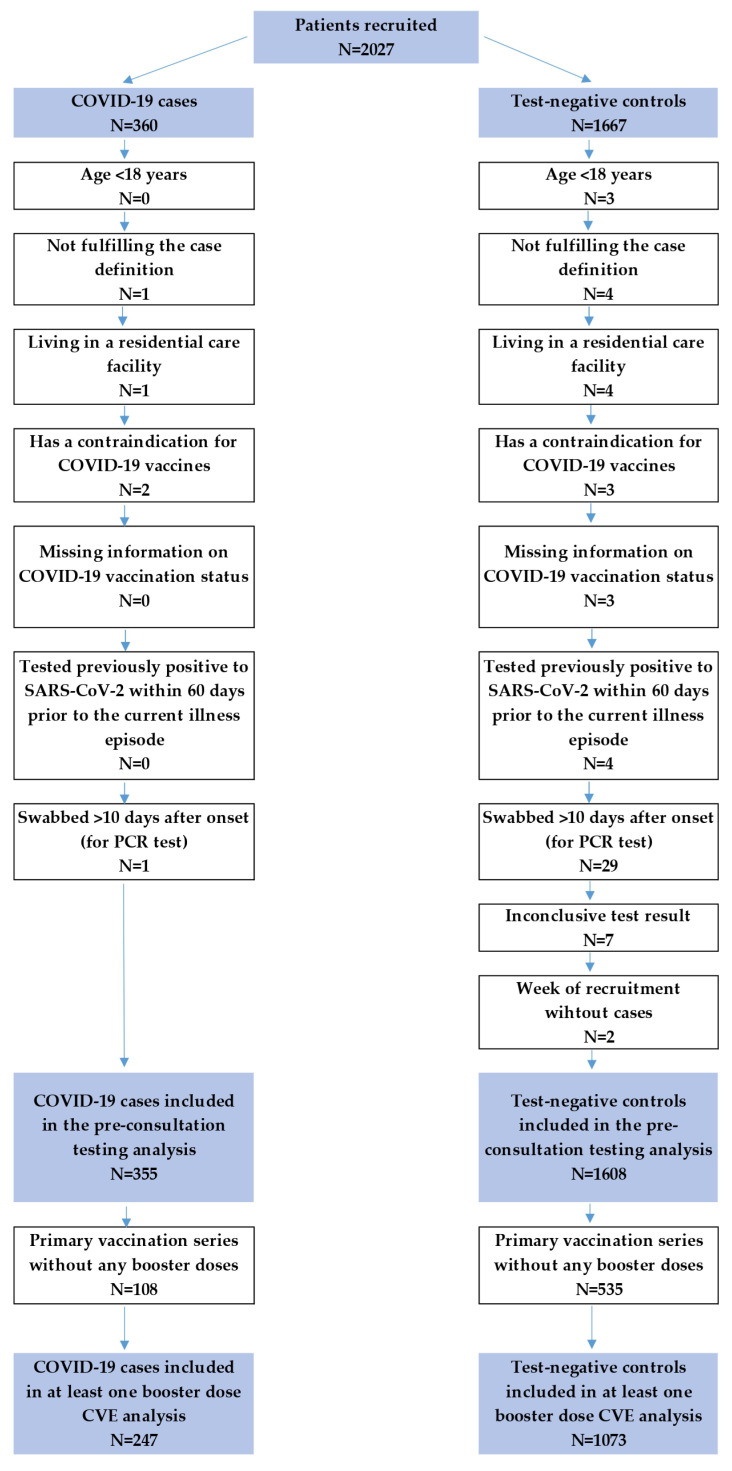
Study eligibility flowchart, primary care-based CVE study, Hungary, September 2022–March 2023.

**Figure 2:**
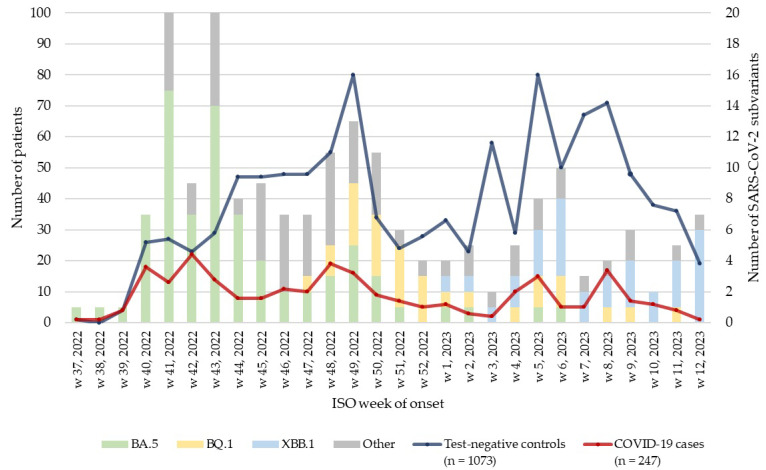
COVID-19 cases and test-negative controls included in the analysis, by week of onset, and the corresponding number of SARS-CoV-2 subvariants, primary care-based CVE study, Hungary, September 2022–March 2023.

A total of 2027 patients were recruited between 11 September 2022 and 24 March, 2023. We excluded 64 patients (5 cases and 59 controls) according to the per-protocol exclusion criteria. The study eligibility flowchart summary is provided in Figure 1. For the “at least one booster” CVE analyses, 1320 patients were included, comprising 247 cases and 1073 controls (Figure 2).

During the recruitment period, a total of 196 SARS-CoV-2-positive samples were sequenced within the study. Of these, 84 (42.9%) belonged to the BA.5 variant group, 32 (16.3%) to the BQ.1 variant group, and 25 (12.8%) to the XBB.1 variant group. The BA.5 subvariant was dominant from week 37 to week 52 of 2022, with 78 out of 135 samples (57.8%) belonging to this group. During the first 12 weeks of 2023, the XBB.1 subvariant became dominant, with 25 out of 61 samples (41.0%) belonging to this group, according to the study results.

Among the participants who met the study eligibility criteria, the median age was slightly higher for cases (53 years, interquartile range [IQR]: 41–68) than for controls (51 years, IQR: 37–66) (Table 1).

The percentage of females was similar between cases (59.1%) and of controls (60.6%). A total of 45.8% of cases and 46.5% of controls reported living with a chronic condition.

The vaccination status assessment was based on the immunization register for 87.8% of the patients, while 5.9% were based on GPs’ electronic medical records and 6.3% was self-reported. At least one booster dose was administered to 76.9% of cases and 78.2% of controls. Among them, 3.7% of cases and 5.4% of controls received the last booster dose within 14–180 days, 34.7% of cases and 30.0% of controls within 181–365 days, and over one year for 61.6% of cases and 64.6% of controls. Among those vaccinated with at least one booster dose, 48.2% (40.0% of cases and 50.1% of controls) received homologous series (only mRNA vaccines) and 51.8% (60.0% of cases and 49.9% of controls) was given heterologous series (not only mRNA vaccines). In this study, 87.8% of heterologous vaccination series (87.7% of cases and 87.8% of controls) included an mRNA booster dose (Supplementary Table S1).

### 3.2. CVE Estimates for “At Least First Booster Vaccination” among 18+ Adults, According to Time since Vaccination and by Age Group

**Figure 3:**
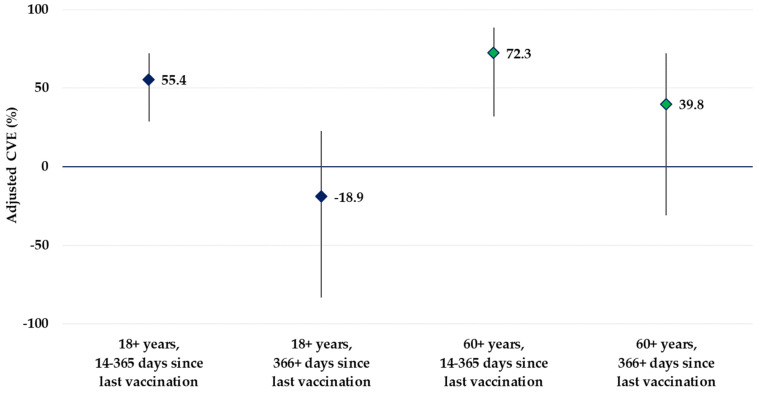
COVID-19 vaccine effectiveness estimates in the 18+- and in the 60+-year-old age groups, according to time since vaccination, primary care-based CVE study, Hungary, September 2022–March 2023.

The first booster CVE was 2.3% (95% confidence interval [CI]: −50.0–36.3%) in the 18–59 age group, and 56.8% (95% CI: 11.9–78.8%) in the 60+ years age group. The CVE was 63.8% (95% CI: −14.0–88.5%) for 14–180 days, and 57.4% (95% CI: 30.5–73.9%) for 181–365 days after vaccination. In addition, the CVE was 55.4% (95% CI: 28.9–72.1%) and −18.9% (95% CI: −83.2–22.8%) for 14–365 and 366+ days after vaccination, respectively. The first booster CVE was 55.4% (95% CI: 28.9–72.1%), 52.9% (95% CI: 15.2–73.9%), and 72.3% (95% CI: 32.0–88.7%) in the 18+, 18–59, and 60+-year-old population vaccinated with the last booster dose within one year, respectively. For those who received their last booster dose more than one year before symptom onset, the first booster CVE was −18.9% (95% CI: −83.2–22.8%) and 39.8% (95% CI: −31.0–72.3%) in the 18+- and 60+-year-old populations, respectively (Figure 3). The detailed CVE results are available in the supplementary tables (Supplementary Tables S2 and S3).

### 3.3. Evaluation of the Protective Effect of Hybrid Immunity against the Circulating Omicron Subvariants, in Comparison to Vaccination or Natural Infection Alone

Of the patients included in the study, 21.9% had a positive SARS-CoV-2 test prior to the current illness episode. Among those, 45.6% had a positive test 60–365 days prior to the current illness episode, while 54.4% had a positive test more than 365 days prior to the current illness episode. The majority of the tests used were rapid antigen tests (65.6%), followed by RT-PCR (31.6%), and 2.8% were unknown. The comparison of overall CVE from the original model with CVE controlled for previous infection also provided no evidence of potential confounding by prior infection in the 18+ age group (CVE = 20.4, 95% CI: −13.9–44.4 and CVE = 20.4, 95% CI: −14.0–44.4, respectively).

**Figure 4:**
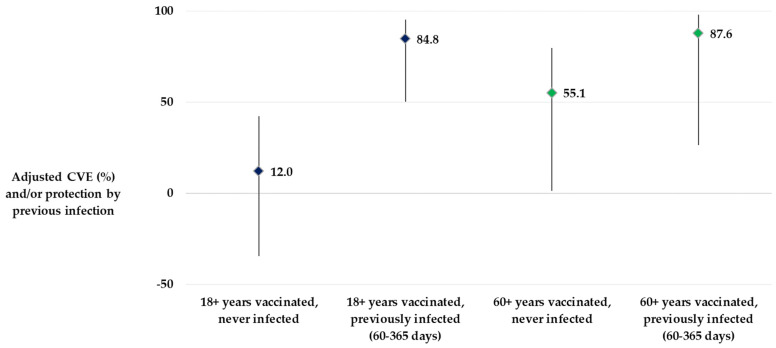
Protection conferred by previous SARS-CoV-2 infection and/or at least first booster vaccination among adults 18 years and older (reference group: had neither previous infection nor vaccination), primary care-based CVE study, Hungary, September 2022–March 2023.

The study found no evidence of protection from self-reported past SARS-CoV-2 infection against the current GP-attended COVID-19 episode, without vaccination. Self-reported past SARS-CoV-2 infection within one year before symptom onset together with “at least one booster” vaccination reduced the risk of GP-attended COVID-19 by 84.8% (95% CI: 50.1–95.4%), 63.0% (95% CI: −28.0–89.3%), and 87.6% (95% CI: 26.4–97.9%) among the 18+, 18–59, and 60+ age groups, respectively, in comparison to those who had neither reported prior infection nor vaccination (see Figure 4 and Supplementary Table S3).

### 3.4. Potential Effects of Testing Prior to the GP Consultation on CVE Estimation

Among the patients, 22.0% had undergone a SARS-CoV-2 test before consulting their GP, with 89.8% of these tests being rapid antigen tests. The prevalence of chronic conditions, self-reported previous SARS-CoV-2 infection, and the number of previous GP consultations was significantly higher among those tested prior to GP consultation, compared to those who had not (Supplementary Table S4). The study found that individuals who had undergone PCT prior to GP consultation were 2.6 times more likely to be a case than those who had not. There was no observed difference between the proportion of persons vaccinated with at least one booster dose among those who underwent PCT and had not (56.9% and 51.1%, respectively, *p*-value 0.755). The comparison of overall CVE from the original model with CVE controlled for previous SARS-CoV-2 test as well provided no evidence of potential confounding by PCT in the 18+ age group (CVE = 20.4, 95% CI: −13.9–44.4 and CVE = 20.3, 95% CI: −14.9–44.7, respectively).

## 4. Discussion

In this study, the estimated CVE was moderate in the 60+ age group, and low in the younger adult age groups against medically attended symptomatic SARS-CoV-2 infections caused by Omicron subvariants, mainly BA.5, BQ.1, and XBB.1. Our data suggest that the protective effect of booster vaccination cannot be demonstrated beyond one year. We found no evidence of protection from previous self-reported COVID-19 against reinfection, without vaccination. A history of SARS-CoV-2 infection with RT-PCR or RAT confirmation within one year prior to symptom onset combined with vaccination increased protection in the 18+ population and provided very high protection in the 60+ population compared to unvaccinated individuals.

Our result regarding higher CVE among the elderly is consistent with a European multicentre study result, in which the first booster VE was 59% (95% CI: 46–69%) in the 50+ age group, and 26% (95% CI: 7–41%) in adults younger than 50 years old [21]. A systematic review and meta-analysis carried out at the time of COVID-19 caused by Omicron variant (B.1.1.529) also found that among participants more than 60 years of age, primary vaccination plus one booster dose decreased the risk of any documented Omicron infection by 57.9% (95% CI: 53.4–62.4%) within 3 months; however, the protection was found to be declined to 14.7% (95%CI: −22.0–51.5%) at six months.

The higher number of contacts and the increased risk of SARS-CoV-2 infection from these contacts in younger age groups may partly explain the lower CVE among them [22,23]. In addition, both previous undiagnosed COVID-19 and COVID-19 vaccine hesitancy may be more prevalent in the young adult population, which could reduce the value of the CVE estimate in the 19–64 age group [24,25,26].

Our study did not demonstrate the protective effect of the booster vaccination more than one year before enrolment in the study. This is consistent with other study results, which suggest that vaccine effectiveness declines over time [3,14,27]. Consistent with other research findings, previous self-reported SARS-CoV-2 infection also did not confer any detectable long-term protection against reinfection in unvaccinated individuals. This suggests rapidly declining protective effect of previous SARS-CoV-2 infection against reinfection as well [14,28].

There are few studies on vaccine effectiveness of different types of vaccine series in different populations. A considerable proportion, 60.0% of cases and 49.9% of controls of our study population (who was vaccinated with at least one booster dose), was administered a heterologous COVID-19 vaccine series. In this study, 87.8% of heterologous vaccination series included an mRNA booster dose. According to a previous comparative analysis, heterologous booster schedules conferred slightly better protection against COVID-19-related outcomes than homologous mRNA vaccine booster schedules [29].

In many countries, obtaining a complete COVID-19 vaccination history, including the date, brand, and vaccine composition for each vaccine dose, is becoming increasingly challenging. It is a strength of our study that the assessment of vaccination status was based on immunization registry in 92.3% of the patients, ensuring completeness and reducing the likelihood of vaccination misclassification becoming a major problem. However, it is worth noting that the study did not recruit any patients who received bivalent mRNA vaccines, which became available in Hungary during the study period [30]. Therefore, it is not possible to calculate the vaccine effectiveness of this type of vaccine.

In this study, we found that 52.2% of the test-negative controls, who represent the source population, had received at least one COVID-19 booster vaccination. This proportion is slightly higher than in the general population aged 18 and older in Hungary (47.5%). One possible explanation for this difference is that the GPs in our study are mainly located in urban areas, where previous study has shown higher vaccination coverage against COVID-19 compared to non-urban areas [31].

In the Omicron era, it is increasingly important to measure whether vaccination can provide additional protection to the existing natural immunity. This study enrolled individuals regardless of previous infection history, and we collected information on their previous SARS-CoV-2 infection with the type of test being specified in 97.2% of patients. The results indicate high CVE among those adults who were both infected with SARS-CoV-2 within one year and had at least one revaccination against COVID-19. This effect was even more pronounced in the elderly population. Similar studies, conducted in countries with higher vaccination coverage and presumably lower levels of natural immunity, have also demonstrated that hybrid immunity confers greater protection than vaccination alone [14,15]. A retrospective cohort study in Singapore also estimated the protection provided by both vaccination and infection against symptomatic reinfection with Omicron subvariants. Similar to the results of our study, it was concluded that previous infection provided minimal protection against reinfection with Omicron subvariants. However, in line with the results of our study, the combination of prior BA.2 infection and booster vaccination with the mRNA vaccine provided a high level of protection against infection with BA.4 or BA.5 variants (protective immunity: 78%; 95% CI: 74–82) and a slightly lower level of protection against reinfection with the XBB variant (protective immunity: 51%; 95% CI 49–53) [32]. Similar conclusion was reported in a review study that pooled the results of vaccine effectiveness and burden of disease studies regarding SARS-CoV-2 Omicron variants [33].

Syndromic outcomes are less specific for COVID-19; thus, VE estimates based on syndromic outcomes are always biased downwards compared with those using laboratory-confirmed outcomes. In line with the WHO guideline, our study used laboratory-confirmed outcome in the analysis, with the preferred method of real-time reverse transcription polymerase chain reaction for laboratory testing of participants, to avoid such bias [5]. However, a limitation of such studies, including ours, is that they may not capture individuals who were infected but did not have a positive PCR or rapid antigen test result and did not seek medical care.

The test-negative method is commonly used to monitor CVE, where patients should be selected in the study based on the clinical case definition alone, without knowledge of the underlying pathogen. The widespread use of RATs during the COVID-19 pandemic may have influenced GP consultations and patient selection. Therefore, it is important to determine whether pre-consultation testing for COVID-19 could influence CVE estimates by affecting GP consultations differentially by vaccination status. In this study, 22.0% of the patients underwent a COVID-19 testing for the current episode, before consulting their GP. These patients had a higher prevalence of chronic disease(s) and more GP consultations in the past, compared to those who did not undergo pre-consultation testing; however, vaccination status and PCT were not associated, and thus PCT was not a confounder in this study. A more in-depth analysis of the potential bias of the pre-consultation testing was not possible due to the small sample size. It is recommended that data collection for this variable be continued in the future for further evaluation of the effect of PCT.

## 5. Conclusions

There is a lack of real-world data on the effectiveness of COVID-19 vaccination from Central and Eastern Europe (CEE) during periods of different circulating Omicron subvariants. The CEE population may be of great interest for CVE research as it has lower vaccination coverage than the EU/EEA average and is likely to have higher natural infection rates. Our study also adds to the literature by providing evidence via a vaccination programme using six different types of vaccines for primary immunization on a population where heterologous immunization was widespread, where mainly mRNA vaccines were used for booster vaccination. The results of this study confirm that booster vaccination provides a significant additional protection, compared to natural immunity, in such a setting and population, especially among older people, who are the main target group for vaccination programmes. It is important that vaccine effectiveness studies continue to be carried out in areas with different vaccination coverage, geography, and social status.

As the acceptance of COVID-19 vaccination declines and the time since last vaccination increases for a growing proportion of the population, booster campaigns should be intensified. It is important to explain in health communications that booster vaccinations also benefit those who have been naturally infected in the past. Clear guidance on when individuals should be vaccinated and emphasis on the benefits of vaccination can help achieve a higher coverage in the population.

Our results are of great importance to public health authorities in planning further vaccination activities and as an example of the continuous monitoring of vaccine effectiveness, which is essential both as a source of scientific evidence and as a way of building trust within the community. Our study may also provide useful information for future research on hybrid immunity.

## Figures and Tables

**Table 1:** Baseline characteristics of SARS-CoV-2 cases (*n* = 247) and test-negative controls (*n* = 1073) in the primary care-based CVE study, Hungary, September 2022–March 2023.

Variables	Number of Laboratory-Confirmed SARS-CoV-2 Cases/Total *n* (%)	Number of Test-Negative Controls/Total *n* (%)
**Age (median [IQR])**	53 [41–68]	51 [37–66]
*Missing*	0	0
**Age group (years)**		
18–59	137/247 (55.47)	693/1073 (64.59)
60+	110/247 (44.53)	380/1073 (35.41)
**Sex**		
Female	146/247 (59.11)	650/1073 (60.58)
*Missing*	0	0
**Symptoms**		
Cough	210/247 (85.02)	864/1073 (80.52)
Shortness of breath	31/247 (12.55)	148/1072 (13.81)
Sore throat	212/247 (85.83)	845/1073 (78.75)
Coryza	209/247 (84.62)	930/1073 (86.67)
**Chronic condition** ^a^		
Presence of chronic condition	113/247 (45.75)	497/1070 (46.45)
No chronic condition	134/247 (54.25)	573/1070 (53.55)
*Missing*	0	3
**Smoking**		
Never or former	191/223 (85.65)	798/1023 (78.01)
Current	32/223 (14.35)	225/1023 (21.99)
*Missing*	24	50
**COVID-19 vaccination status**		
**Unvaccinated**		
All	57/247 (23.08)	234/1073 (21.81)
18–59 years (age)	40/137 (29.20)	205/693 (29.58)
60+ years (age)	17/110 (15.45)	29/380 (7.63)
**Full primary series plus at least one booster dose**		
All	190/247 (76.92)	839/1073 (78.19)
18–59 years (age)	97/137 (70.80)	488/693 (70.42)
60+ years (age)	93/110 (84.55)	351/380 (92.37)

^a^ The presence of chronic condition is defined as reporting at least one of the following conditions: diabetes, heart disease, hypertension, lung disease, immunodeficiency, cancer, or renal disease.

## Data Availability

The original contributions presented in the study are included in the article; further inquiries can be directed to the corresponding author.

## Supplementary Materials

The following supporting information can be downloaded at: https://www.mdpi.com/article/10.3390/vaccines12050496/s1, Supplementary Table S1. Baseline characteristics of SARS-CoV-2 cases and test-negative controls included in at least one booster CVE analysis, primary care-based CVE study, Hungary, September 2022–March 2023. Supplementary Table S2. COVID-19 vaccine effectiveness estimates for at least first-booster vaccination among 18+ adults, primary care-based CVE study, Hungary, September 2022–March 2023. Supplementary Table S3. Protection conferred by prior infection and/or COVID-19 vaccine effectiveness for at least first-booster vaccination among 18+ adults (reference group: no prior infection and no vaccination), primary care-based CVE study, Hungary, September 2022–March 2023. Supplementary Table S4. Baseline characteristics of those who had previous SARS-CoV-2 test and those without previous SARS-CoV-2 test, primary care-based CVE study, Hungary, September 2022–March 2023.
