# Supplementary material for: Vaccine Effectiveness against GP-Attended Symptomatic COVID-19 and Hybrid Immunity among Adults in Hungary during the 2022–2023 Respiratory Season Dominated by Different SARS-CoV-2 Omicron Subvariants
Source: Vaccines (Basel). 2024 May 4;12(5):496. doi: 10.3390/vaccines12050496 (PMC11125656; doi:10.3390/vaccines12050496)
Supplement: Supplementary file 1 [file vaccines-12-00496-s001.zip › vaccines-2935328-supplementary.pdf]

vaccines

MDPI

Supplement

Supplement Table S1. Baseline characteristics of SARS-CoV-2 cases and test-negative controls included in at least one booster CVE analysis, VEBIS primary care study, Hungary, September 2022 -March 2023.

Characteristics	18+ included in at least first booster analyses (Full primary series + at least 1 booster VS. Unvaccinated)				
	SARS-CoV-2 cases (n = 247)		Test-negative controls (n = 1,073)		p-value
	n	%	n	%	
**Age (median [IQR])**	53 [41–68]		51 [37–66]		0.015
**Missing**	0	0.00	0	0.00	
**Age group (years)**					
**18-59**	137	55.47	693	64.59	
**60+**	110	44.53	380	35.41	0.007
**Sex**					
**Female**	146	59.11	650	60.58	
**Male**	101	40.89	423	39.42	0.671
**Missing**	0	0.00	0	0.00	
**BMI** [a]					
**max. 29**	172	69.64	736	68.59	
**30+**	75	30.36	337	31.41	0.750
**Missing**	0	0.00	0	0.00	
**Symptoms**					
**Cough**	210	85.02	864	80.52	0.102
**Short breath**	31	12.55	148	13.81	0.604
**Sore throat**	212	85.83	845	78.75	0.012
**Coryza**	209	84.62	930	86.67	0.397
**Fever**	167	67.61	603	56.20	0.001
**Malaise**	222	89.88	860	80.15	0.000
**Myalgia**	166	67.21	534	49.77	0.000
**Headache**	171	69.23	600	55.97	0.000
**Anosmia**	17	6.88	28	2.61	0.001
**Ageusia**	14	5.67	24	2.24	0.004
**Chronic condition** [ba]					
**Presence of chronic condition**	69	27.94	278	25.91	0.480
**No chronic condition**	174	70.45	784	73.07	
**Missing**	4	1.62	11	1.03	
**Chronic condition** [bb]					
**Presence of chronic condition**	113	45.75	497	46.32	0.842

**No chronic condition**	134	54.25	573	53.40	
**Missing**	0	0.00	3	0.28	
**Severity [c]**					
**0**	231	93.52	979	91.24	
**1+**	16	6.48	94	8.76	0.242
**Missing**	0	0.00	0	0.00	
**GP consultation [d]**					
**0-1**	27	10.93	145	13.51	
**2+**	220	89.07	928	86.49	0.277
**Missing**	0	0.00	0	0.00	
**0-4**	102	41.30	505	47.06	
**5+**	145	58.70	568	52.94	0.101
**Missing**	0	0.00	0	0.00	
**Smoking [e]**					
**Never or former**	191	77.33	798	74.37	
**Current**	32	12.96	225	20.97	0.011
**Missing**	24	9.72	50	4.66	
**Swab delay [f]**					
**max. 2 days**	119	48.18	630	58.71	
**3+ days**	128	51.82	443	41.29	0.003
**Missing**	0	0.00	0	0.00	
**Previous SARS-CoV-2 test [g]**					
**Previous test**	98	39.68	215	20.04	0.000
**No previous test**	149	60.32	858	79.96	
**Missing**	0	0.00	0	0.00	
**Self-reported prior SARS-CoV-2 infection [h]**					
**Prior**	45	18.22	225	20.97	0.334
**No prior**	202	81.78	848	79.03	
**Missing**	0	0.00	0	0.00	
**Self-reported prior SARS-CoV-2 infection [h]**					
**Prior (60-365 days)**	13	6.05	109	11.39	0.020
**No prior**	202	93.95	848	88.61	
**Self-reported prior SARS-CoV-2 infection [h]**					
**Prior (366+ days)**	32	13.68	116	12.03	0.494
**No prior**	202	86.32	848	87.97	
**COVID-19 vaccination status**					
**Unvaccinated**					
**All**	57	23.08	234	21.81	
**18-59 years (age)**	40	29.20	205	29.58	
**60+ years (age)**	17	15.45	29	7.63	

**Full primary series plus at least one booster dose**					
**All**	190	76.92	839	78.19	0.664
**18-59 years (age)**	97	70.80	488	70.42	0.928
**60+ years (age)**	93	84.55	351	92.37	0.013
**14-180 days (time since last vaccination)**	7	2.83	45	4.19	0.296
**181-365 days (time since last vaccination)**	66	26.72	252	23.49	0.720
**366+ days (time since last vaccination)**	117	47.37	542	50.51	0.501
**Homologous series** [i]	76	30.77	420	39.14	0.123
**Heterologous series** [i]	114	46.15	419	39.05	0.542

[a] BMI calculated as weight/(height*height).

[ba] The presence of chronic condition is defined as reporting at least one of the following conditions: diabetes, heart disease, lung disease, immunodeficiency.

[bb] The presence of chronic condition is defined as reporting at least one of the following conditions: diabetes, heart disease, hypertension, lung disease, immunodeficiency, cancer, renal disease.

[c] Severity: number of hospitalizations in the previous 12 months for chronic disease.

[d] Number of GP consultations in the previous 12 months.

[e] Former: stopped smoking at least 1 year before inclusion in the study. Current smoker: any smoking can be included: cigarettes, cigars, vaping, etc.

[f] Swab delay: number of days between date of swab and date of onset.

[g] Previous SARS-CoV-2 test: Has the patient had a previous SARS-CoV-2 test (e.g. PCR, antigen, both, unknown) related to his/her current symptoms?

[h] Previous SARS-CoV-2 infection: Has the patient had a positive SARS-CoV-2 test prior to this illness episode? (At all, 60-365, 366+ days before current onset)

[i] Homologous series: primary series and at least one dose booster dose are all mRNA vaccines. Heterologous series: primary series and at least one dose booster are not all mRNA vaccines.

Supplement Table S2. COVID-19 vaccine effectiveness estimates for at least first booster vaccination among 18+ adults, VEBIS primary care study, Hungary, September 2022 -March 2023.

Population	Time since last booster vaccination (days)	Adjusted CVE (95% CI)*	N	Vacci-nated cases	Unvacci-nated cases	Vacci-nated controls	Unvacci-nated controls
Adults aged 18 years and above	14+	20.4 (-13.9; 44.4)	1,320	190	57	839	234
Adults aged 18 years and above (plus adjustment for previous SARS-CoV-2 infection)	14+	20.4 (-14.0; 44.4)	1,320	190	57	839	234
Adults aged 18 years and above (plus adjustment for previous SARS-CoV-2 test)	14+	20.3 (-14.9; 44.7)	1,320	190	57	839	234
Adults aged 18 years and above reporting no previous SARS-CoV-2 test	14+	29.5 (-9.4; 54.6)	1,007	112	37	671	187
Adults aged 18 years and above reporting previous SARS-CoV-2 test	14+	10.5 (-114.6; 43.1)	313	78	20	168	47
Adults aged 18-59 years	14+	2.3 (-50.0; 36.3)	830	97	40	488	205
Adults aged 18-59 years (plus adjustment for previous SARS-CoV-2 infection)	14+	1.4 (-51.5; 35.8)	830	97	40	488	205
Adults aged 18-59 years and above (plus adjustment for previous SARS-CoV-2 test)	14+	0.7 (-53.8; 35.9)	830	97	40	488	205
Adults aged 60 years and above	14+	56.8 (11.9; 78.8)	490	93	17	351	29
Adults aged 60 years and above (plus adjustment for previous SARS-CoV-2 infection)	14+	57.7 (13.3; 79.4)	490	93	17	351	29
Adults aged 60 years and above (plus adjustment for previous SARS-CoV-2 test)	14+	58.3 (14.2; 79.7)	490	93	17	351	29

Adults aged 18 years and above	14-180	63.8 (-14.0; 88.5)	343	7	57	45	234
Adults aged 18 years and above	181-365	57.4 (30.5; 73.9)	609	66	57	252	234
Adults aged 18 years and above	14-365	55.4 (28.9; 72.1)	661	73	57	297	234
Adults aged 18 years and above (plus adjustment for previous SARS-CoV-2 infection)	14-365	54.6 (27.4; 71.6)	661	73	57	297	234
Adults aged 18 years and above (plus adjustment for previous SARS-CoV-2 test)	14-365	55.9 (29.0; 72.6)	661	73	57	297	234
Adults aged 18 years and above reporting no previous SARS-CoV-2 test	14-365	57.6 (24.2; 76.3)	488	38	37	226	187
Adults aged 18 years and above reporting previous SARS-CoV-2 test	14-365	51.5 (-16.5; 79.8)	173	35	20	71	47
Adults aged 18 years and above	366+	-18.9 (-83.2; 22.8)	950	117	57	542	234
Adults aged 18 years and above (plus adjustment for previous SARS-CoV-2 infection)	366+	-19.1 (-83.5; 22.7)	950	117	57	542	234
Adults aged 18 years and above (plus adjustment for previous SARS-CoV-2 test)	366+	-20.0 (-86.0; 22.5)	950	117	57	542	234
Adults aged 18-59 years	14-365	52.9 (15.2; 73.9)	461	38	40	178	205
Adults aged 60 years and above	14-365	72.3 (32.0; 88.7)	200	35	17	119	29
Adults aged 18-59 years	366+	-85.7 (-225.5; -5.9)	614	59	40	310	205
Adults aged 60 years and above	366+	39.8 (-31.0; 72.3)	336	58	17	232	29

* adjusted for symptom onset week, age, sex, and presence of at least one chronic condition including diabetes, immunodeficiency, lung disease, and heart disease
CVE: COVID-19 vaccine effectiveness
CI: confidence interval

Supplement Table S3. Protection conferred by prior infection and/or vaccine effectiveness COVID-19 vaccine effectiveness for at least first booster vaccination among 18+ adults (reference group: no prior infection and no vaccination), VEBIS primary care study, Hungary, September 2022 -March 2023.

Population	Time since last booster vaccination (days)	Adjusted CVE (95% CI)*	N	Vaccinated cases	Unvaccinated cases	Vaccinated controls	Unvaccinated controls
Adults aged 18 years and above unvaccinated, previously infected (60-365 days) VS. unvaccinated, never infected	14+	-246.1 (-929.3; -16.4)	244	8	40	20	176
Adults aged 18 years and above unvaccinated, previously infected (366+ days) VS. unvaccinated, never infected	14+	-210.8 (-787; -8.9)	263	9	40	38	176
Adults aged 18 years and above vaccinated, never infected VS. unvaccinated, never infected	14+	12.0 (-34.4; 42.4)	1,050	162	40	672	176
Adults aged 18 years and above vaccinated, previously infected (60-365 days) VS. unvaccinated, never infected	14+	84.8 (50.1; 95.4)	310	5	40	89	176
Adults aged 18 years and above vaccinated, previously infected (366+ days) VS. unvaccinated, never infected	14+	-45.3 (-191.6; 27.6)	317	23	40	78	176
Adults aged 18-59 years unvaccinated, previously infected (60-365 days) VS. unvaccinated, never infected	14+	-367 (-1395.1; -45.9)	203	8	25	17	153
Adults aged 18-59 years unvaccinated, previously infected (366+ days) VS. unvaccinated, never infected	14+	-212.4 (-882.2; 0.6)	220	7	25	35	153
Adults aged 18-59 years vaccinated, never infected VS. unvaccinated, never infected	14+	-18.6 (-99.0; 29.3)	620	75	25	367	153

Adults aged 18-59 years vaccinated, previously infected (60-365 days) VS. unvaccinated, never infected	14+	63.0 (-28.0; 89.3)	244	4	25	62	153
Adults aged 18-59 years vaccinated, previously infected (366+ days) VS. unvaccinated, never infected	14+	-108.1 (-382.9; 10.3)	255	18	25	59	153
Adults aged 60 years and above unvaccinated, previously infected (60-365 days) VS. unvaccinated, never infected	14+	49.5 (-1307.0; 98.2)	41	0	15	3	23
Adults aged 60 years and above unvaccinated, previously infected (366+ days) VS. unvaccinated, never infected	14+	-54.8 (-2388.4; 90.4)	43	2	15	3	23
Adults aged 60 years and above vaccinated, never infected VS. unvaccinated, never infected	14+	55.1 (1.2; 79.6)	430	87	15	305	23
Adults aged 60 years and above vaccinated, previously infected (60-365 days) VS. unvaccinated, never infected	14+	87.6 (26.4; 97.9)	66	1	15	27	23
Adults aged 60 years and above vaccinated, previously infected (366+ days) VS. unvaccinated, never infected	14+	39.0 (-138.4; 84.4)	62	5	15	19	23

* adjusted for symptom onset week, age, sex, and presence of at least one chronic condition including diabetes, immunodeficiency, lung disease, and heart disease
CVE: COVID-19 vaccine effectiveness (When comparing unvaccinated patients, the measure of effect is the protection conferred by infection rather than vaccine effectiveness.)
CI: confidence interval

Supplement Table S4. Baseline characteristics of those had previous SARS-CoV-2 test and those without previous SARS-CoV-2 test, VEBIS primary care study, Hungary, September 2022 -March 2023.

Characteristics	Previous SARS-CoV-2 test VS. No previous SARS-CoV-2 test				
	Previous SARS-CoV-2 test (n = 432)		No previous SARS-CoV-2 test (n = 1,531)		p-value
	n	%	n	%	
**Age (median [IQR])**	49 [35–64.5]		48 [34–63]		0.155
**Missing**	0	0.00	0	0.00	
**Age group (years)**					
**18-59**	288	66.67	1064	69.50	
**60+**	144	33.33	467	30.50	0.262
**Sex**					
**Female**	268	62.04	904	59.05	
**Male**	164	37.96	627	40.95	0.263
**Missing**	0	0.00	0	0.00	
**BMI** [a]					
**max. 29**	300	69.44	1,060	69.24	
**30+**	132	30.56	471	30.76	0.934
**Missing**	0	0.00	0	0.00	
**Chronic condition** [ba]					
**Presence of chronic condition**	128	29.63	328	21.42	0.000
**No chronic condition**	301	69.68	1,185	77.40	
**Missing**	3	0.69	18	1.18	
**Chronic condition** [bb]					
**Presence of chronic condition**	201	46.53	591	38.60	0.003
**No chronic condition**	231	53.47	936	61.14	
**Missing**	0	0.00	4	0.26	
**Severity** [c]					
**0**	399	92.36	1,419	92.68	
**1+**	33	7.64	112	7.32	0.820
**GP consultation** [d]					
**0-1**	48	11.11	235	15.35	0.027
**2+**	384	88.89	1,296	84.65	
**Missing**	0	0.00	0	0.00	
**0-4**	178	41.20	759	49.58	0.002
**5+**	254	58.80	772	50.42	
**Missing**	0	0.00	0	0.00	

**Smoking [e]**					
**Never or former**	313	72.45	1,126	73.55	
**Current**	82	18.98	340	22.21	0.305
**Missing**	37	8.56	65	4.25	
**Swab delay [f]**					
**max. 2 days**	243	56.25	873	57.02	0.775
**3+ days**	189	43.75	658	42.98	
**Missing**	0	0.00	0	0.00	
**Case status**					
**SARS-CoV-2 case**	132	30.56	223	14.57	0.000
**Test-negative control**	300	69.44	1,308	85.43	
**Missing**	0	0.00	0	0.00	
**Self-reported prior SARS-CoV-2 infection [g]**					
**Prior**	134	31.02	296	19.33	0.000
**No prior**	298	68.98	1,235	80.67	
**Missing**	0	0.00	0	0.00	
**Self-reported prior SARS-CoV-2 infection [g]**					
**Prior (60-365 days)**	70	19.02	126	9.26	0.000
**No prior**	298	80.98	1,235	90.74	
**Self-reported prior SARS-CoV-2 infection [g]**					
**Prior (366+ days)**	64	17.68	170	12.10	0.005
**No prior**	298	82.32	1,235	87.90	
**COVID-19 vaccination status**					
**Unvaccinated**					
**All**	67	15.51	224	14.63	
**18-59 years (age)**	56	19.44	189	17.76	
**60+ years (age)**	11	7.64	35	7.49	
**Full primary series plus at least one booster dose**					
**All**	246	56.94	783	51.14	0.755
**18-59 years (age)**	137	47.57	448	42.11	0.861
**60+ years (age)**	109	75.69	335	71.73	0.924
**14-180 days (time since last vaccination)**	18	4.17	34	2.22	0.075
**181-365 days (time since last vaccination)**	88	20.37	230	15.02	0.188
**366+ days (time since last vaccination)**	140	32.41	519	33.90	0.540
**Homologous series [h]**	130	30.09	366	23.91	0.319
**Heterologous series [h]**	116	26.85	417	27.24	0.677

[a] BMI calculated as weight/(height*height).

[ba] The presence of chronic condition is defined as reporting at least one of the following conditions: diabetes, heart disease, lung disease, immunodeficiency.
[bb] The presence of chronic condition is defined as reporting at least one of the following conditions: diabetes, heart disease, hypertension, lung disease, immunodeficiency, cancer, renal disease.
[c] Severity: number of hospitalizations in the previous 12 months for chronic disease.
[d] Number of GP consultations in the previous 12 months.
[e] Former: stopped smoking at least 1 year before inclusion in the study. Current smoker: any smoking can be included: cigarettes, cigars, vaping, etc.
[f] Swab delay: number of days between date of swab and date of onset.
[g] Previous SARS-CoV-2 infection: Has the patient had a positive SARS-CoV-2 test prior to this illness episode? (At all, 60-365, 366+ days before current onset)
[h] Homologous series: primary series and at least one dose booster dose are all mRNA vaccines. Heterologous series: primary series and at least one dose booster are not all mRNA vaccines.
